# Editorial: Mathematical Modeling of Cardiovascular Systems: From Physiology to the Clinic

**DOI:** 10.3389/fphys.2019.01259

**Published:** 2019-09-27

**Authors:** Ghassan Kassab, Julius Guccione

**Affiliations:** ^1^California Medical Innovations Institute, San Diego, CA, United States; ^2^Department of Surgery, University of California, San Francisco, San Francisco, CA, United States

**Keywords:** biomechanics, cardiac mechanics, vascular mechanics, heart failure, coronary circulation

Biomedical research has enjoyed an eruption of reductionism where organs are reduced to their respective tissues, cells, and molecules. Although reductionism (including proteomic and genomic) is a powerful approach to provide information on the components of the system, integration of the components and their interactions are necessary to reveal organ phenotype and function (Kassab et al., 2016; Heikhmakhtiar and Lim, 2018). Biological integration is inherently complex and requires mathematical “gluing” of numerous components of the system with numerous variables. Physics-based mathematical modeling is well-tailored for the task where the number of variables is so great that it eludes intuition. The past several decades have enjoyed enormous advancements in mathematical modeling as computational capabilities have advanced substantially and experimental databases to inform and calibrate the computational models have become available to minimize the number of *ad hoc* assumptions. Applications of mathematical models have ranged from understanding basic biological systems to generate new hypotheses to patient-specific modeling and simulation studies that can aid medical diagnosis and help design optimal treatments (Wenk et al., 2009). Modeling and simulation studies have an important role here because they provide knowledge and insights not available from existing data modalities and make evidence-generation more efficient by reducing the number of real patients needed in clinical studies. Furthermore, the utility of mathematical models remains largely removed from the clinic. The objective of this issue of “Frontiers in Physiology” is to place the spotlight on computational integration with emphasis on validation and clinical translation of mathematical models.

The cardiovascular system which includes the heart and blood vessels has enjoyed prodigious efforts on both reduction and integration for understanding physiology and pathophysiology. For example, it is now clinical practice to compute pressure drop in the coronary arteries using computerized tomography (to determine fractional flow reserve; Tan et al., 2017), and non-invasively measure regional heart wall motion and strain in patients by using echocardiography or cardiac magnetic resonance imaging, in order to solve the inverse problem and quantify regional myocardial diastolic compliance and systolic contractility. On the latter, there still is no reliable force transducer or strain gauge for directly measuring forces or stresses in the intact heart wall without interfering with heart function. Clinicians, engineers, and physiologists have been interested in measurement and/or computation of heart wall stress since the early 1900s. Myocardial stress is an important boundary condition for coronary blood flow, and it is directly related to myocardial oxygen consumption (Yin, 1981). The working hypothesis is that early and late cardiovascular disease treatment outcomes will be improved by using surgical and/or percutaneous techniques that reduce forces or stresses to homeostatic levels (Grossman, 1980). Since regional heart wall stress cannot be measured reliably, mathematical modeling based on the conservation laws of continuum mechanics is needed. Because heart wall geometry (including its fibrous architecture) is fully 3D and the mechanical properties of beating myocardium are non-linear, there are no exact solutions of the governing differential equations of motion. Thus, numerical methods are required to find numerical approximations. The most versatile numerical method for simulation of cardiovascular system is the finite element (FE) method (Guccione et al., 2010), which is widely used in the automotive and aerospace industries. Most FE models concerned with heart disease include only the left ventricular (LV) heart chamber because it is under the greatest stress (highest pressures) and thus, most prone to failure.

Currently, numerous computational models can simulate the behavior of the LV, a few models can simulate both ventricles, and even fewer can simulate all full four-chambered heart (Baillargeon et al., 2014). The response of FE model is governed by realistic electrical, structural, and fluid flow physics. Significant recent improvements have been made in determination of diastolic (Sack et al.) and systolic (Sack et al., 2016) myocardial material properties in FE models of normal human LV (Genet et al., 2014). Relatively few computational studies have investigated the effects of mechanical circulatory support devices on cardiac function using realistic geometries (Lim et al., 2012; McCormick et al., 2013). A recent study of the effects of an LV assist device on acute left heart failure (Sack et al., 2016) presented stress results in both ventricles.

Demands for medical device evidence development addressing patient safety, therapeutic efficacy, and cost-benefit determination are increasing. These demands are well-documented drivers of medical device clinical trial size, complexity, timelines, and costs. At the same time, and seemingly paradoxically, patient and provider demands for faster treatment access, and more innovative treatment options are increasing. Given the legitimacy of these stakeholders' data demands, combined with the realities of resource limitations (budget and time), historic data acquisition techniques will soon no longer meet patient and provider needs. While numerous solutions have been hypothesized, the adoption of clinical trial solutions which make clinical trials more efficient and provide the data necessary to show safety and efficacy has been scarce. Modeling and simulation add value to the medical device innovation ecosystem by providing knowledge and insights not available from existing data modalities and making evidence generation more efficient by reducing the need for more burdensome types of data and information. We can more fully utilize modeling and simulation to minimize burden on patients, investigators, manufacturers and regulators, with mechanisms that support efficient review of novel products and approaches without compromising safety or effectiveness. “In the future, computer-based modeling may change the way we think about device validation in other ways, allowing for much smaller clinical trials, …, in that some ‘clinical' information may be derived from simulation (Faris and Shuren, 2017).” Stated another way, digital evidence (evidence from well-controlled modeling and simulation studies) could serve as “clinical” information to support the evaluation of medical devices.

It is our hope that this edition of “Frontiers in Physiology” titled “Mathematical Modeling of Cardiovascular Systems: From Physiology to the Clinic” will serve to highlight powerful analytical tools that may advance to the clinic to improve safety and efficacy of existing treatments and advance novel diagnostics and therapeutics. In this spirit, we are delighted to include 23 articles contributed by 101 authors to this special issue. The topics range from models of cardiac and vascular system in health and disease from molecular to the organ system including simulations of cardiovascular devices. To promote these and other translational and transformational efforts, it is important to encourage further computational modeling that range from molecular, to cellular, to tissues and organs in partnership with experimental validations. Validation is key to this effort as it will boost confidence in the computational simulations that will translate from the academic laboratories to the clinic.

## Author Contributions

All authors listed have made a substantial, direct and intellectual contribution to the work, and approved it for publication.

### Conflict of Interest

The authors declare that the research was conducted in the absence of any commercial or financial relationships that could be construed as a potential conflict of interest.
